# Effect of photo-bio-modulation on pain perception with inferior alveolar nerve block among children: A split mouth study

**DOI:** 10.6026/973206300210914

**Published:** 2025-05-31

**Authors:** Manager Farah, Barjatya Khushboo, Malik Murtuza, Vankar Nidhi, Garg Deepti

**Affiliations:** 1 Department of Pediatric and Preventive Dentistry, Sri Aurobindo College of Dentistry, Indore, India; 2 Department of Oral and Maxillofacial Surgery, Private practitioner, Indore, India; 3 Department of Physiotherapy, Sri Aurobindo college of Paramedical and Allied Sciences, Indore, India

**Keywords:** Low-level light therapy, pediatric dentistry, pain management, laser, analgesia

## Abstract

Local anesthesia is crucial when it comes to pain relief in dental treatment. However, needle insertion often causes non-compliance in children. Therefore, it is of interest to assess the effect of 940-nm laser photobiomodulation on pain while administering inferior alveolar nerve block (IANB) among children between 8-12 years of age. Photobiomodulation pain was assessed using the Wong-baker faces pain rating scale, face, legs, activity, cry and consolability (FLACC) scale and pulse oximeter. Results show that photobiomodulation using a 940-nm laser reduced pain during injection of IANB in children between 8 to 12 years of age.

## Background:

Pain is "an unpleasant emotional and sensory experience associated with actual or potential tissue damage or described in terms of such damage". Minimizing distress and pain is crucial for pediatric dentists, as pain-free needle insertion enhances trust, co-operation and treatment compliance [1]. Low level laser therapy, nowadays named photobiomodulation is a type of non-thermal light treatment that has demonstrated positive effects in reducing pain and inflammation while modulating immune responses [2]. Some of its applications include reduction of gag reflex [3] reduction of anxiety in pediatric patients enhancing soft tissue healing post extraction [4] alleviating Temporomandibular joint pain bactericidal and hemostatic effect [5, 6]. Therefore, it is of interest to assess the photobiomodulation impact of 940-nm laser on the pain perception at the time of IANB in 8- to 10-year-old children.

## Methodology:

This research is a double-blinded, split-mouth, randomized controlled trial conducted after obtaining approval from the Ethical and Research Committee, SAIMS. (IEC NO: SAIMS/IEC/RC/40/24). The software G*Power version 3.1.9.7 was used for calculating the sample size. Values were taken from a review of previous literature for estimation of the effect size and power of the study [7]. At 80% power and the margin of error 5%, the value obtained was twenty. So, each study group comprised of twenty samples. The study included patients aged 8-12 years requiring bilateral IANB for a dental procedure, with written parental consent, Frankl scale score of 3 or 4 and no systemic diseases or bleeding disorders. Patients on medications affecting anesthetic assessment, those with active pathosis at the injection site, known allergies to local anesthesia, special needs were excluded from the study.

## Blinding and allocation:

The examiner and the patients assessing scores of pain and analyzing the data were unaware of groups assigned and whether the laser was set to on or off mode. A separate researcher, also blinded to the laser's operational mode, completed the pain scale checklist. Additionally, the statistician received the data labeled as group I and group II, with no details provided about what intervention was assigned to each group. A total of twenty patients requiring IANB were selected as per the inclusion and exclusion criteria. Quadrants of the patients were randomly assigned to Group 1 (Photobiomodulation) or Group 2 (Placebo). Injection site was dried and 2% lignocaine gel was applied for 30 seconds. The first ten patients received photobiomodulation (PBM) for 20 seconds on one side during their first visit, followed by IANB and placebo (machine off) on the contralateral side during their second visit, also followed by IANB. The next ten patients received placebo first, followed by IANB and photobiomodulation during their second visit. A 1-week interval was used between visits to minimize the impact of previous needle prick experiences 1.8 ml of Lignocaine hydrochloride and adrenaline 1:2000000 (Themicaine AD, Themis Medicare limited, Hardware, Uttarakhand, India) along with a26-gaugeneedle (Hindustan syringes and medical devices Ltd, Maharashtra) was used for the injection [7]. Patients were subjected to Photobiomodulation (Tech Laser 302, Technomed Electronics) with a 940nm [3, 4 and 12] wavelength, 200 mW power in continuous wave, 200 mW/cm2 , 4-6 J/cm2 power density and energy density, respectively. A hand-piece with 1 cm2 spot size for 20 seconds at a 2-mmdistance was used for exposure [2]. In the control group, following the application of a topical anesthetic, the device was positioned for duration of 20 seconds with the laser turned off. To ensure safety, the child and the operator both wore protective eyewear during the procedure.

## Assessment:

For subjective assessment, the patient (who was blinded) described his/her pain by pointing to the Wong-Baker Faces pain rating Scale. For objective assessment, the pain was assessed by an investigator (who was blinded) using the Face, Legs, Activity, Cry and Consolability (FLACC scale). Pulse oximeter was used to assess the heart rate [1, 7, 8].

## Statistical analysis:

MS Excel 2010was used for entering data and SPSS software version 21.0 was used for analysis. It was presented in a frequency table. Descriptive statistics were calculated as mean and Standard Deviation for quantitative variable and frequency and percentage for qualitative or categorical variable. Bar diagram was used for qualitative variables. For Inter-group comparison Independent 't' test was used. P value <.05 was considered statistically significant. Confidence interval was set at 95%.

## Discussion:

The results revealed that low-level laser therapy group has a lower mean the Wong-baker faces pain rating scale score (3.00) compared to the Placebo group, suggesting that low level laser therapy might be more efficacious in reducing pain as measured by the Wong-baker faces pain rating scale. The low-level laser therapy group also has a lower mean FLACC score (1.30) compared to the Placebo group (2.60), indicating less discomfort or pain in the low-level laser therapy group with statistically significant results (Table 1). The mean pulse oximeter reading is slightly lower in the low-level laser therapy group (84.63) compared to the Placebo group (90.10), but both groups have similar ranges and distributions with a statistically non-significant result. Intergroup comparison using't' test revealed both the wong-baker faces rating scale and FLACC Scale scores show significant differences (p < 0.05), indicating low level laser therapy group experienced lower pain levels compared to the Placebo group. The Pulse Oximeter readings in contrast reveal no significant differences (p > 0.05), suggesting no measurable difference of low-level laser therapy on oxygen saturation or pulse rate between the two groups. This double-blinded, split-mouth, randomized controlled trial showed the effect of low-level laser therapy on pain perception in child ren while administration of LA. Photobiomodulation uses light energy to elicit biological responses from cell. Factors such as enhancing lymphatic flow, circulation, fibroblast activity and endorphin release while reducing nerve depolarization and inflammation, contribute to its clinical effects [9]. However, the efficacy of photobiomodulation and standard protocols remains unclear due to variations in irradiation parameters, injection sites, pain assessment methods and study populations [2]. Our results showed higher pain and discomfort in the placebo group for IANB, as indicated by the Wong-baker faces pain rating scale and FLACC scores. Pulse oximeter readings were similar, but slightly higher in the low-level laser therapy group, suggesting its potential effectiveness in reducing pain and discomfort. According to "optical window," Non Infra-red wavelengths can penetrate deep through dental hard structures to reach the pulp tissue, causing biological effects.

Desired wavelengths for therapeutic effects are typically in 600-1000 nm range, in red to near infra-red spectrum with energy density up to 10 J/cm^2^, effectively stimulating biological processes [9]. Like our study, Dehgan *et al.* evaluated the different energy densities and powers of 940-nm photobiomodulation like 0.3 W, 0.4 W and 0.5 W with 20-second irradiation corresponding to 69 J/cm^2^, 92 J/cm^2^ and 115 J/cm^2^, respectively on pain perceptions during local anesthesia injection on the buccal surface of mandibular and maxillary primary first molars and found statistically significant results with photobiomodulation+10% Lidocaine topical anesthetic application reducing pain [10]. Contrary to our results, Ghabrei *et al.* evaluated effect of the simultaneous application of 960 nm photobiomodulation at 100 mW, 4 J/cm^2^ with benzocaine anesthetic gels on pain during injection into buccal mucosa in an adult. There was no clinically significant difference between the photobiomodulation and control groups [11]. In this study, the combined impact of photobiomodulation and local anesthetic gel, which is commonly used in dental procedures of pediatric patients, was evaluated. Our aim was to assess the enhanced benefits of incorporating photobiomodulation into this approach. The literature shows a maximum application time of 3 minutes.

Jagtap *et al.* [12] used 3 minutes of photobiomodulation at a power of 60 mW and Sattayut *et al.* [13] for 2 minutes at 30 mW powers and 27.69 J/^2^ energy intensity. Jagtap *et al.* reported positive results a negative result was observed by Sattayut *et al.* But both studies had some missing parameters. In studies where the energy density was reported as 300 mW, the application time was twenty seconds except in a study by Varshini *et al.* where only a 1-minute application using 300 mW power was reported [14. Their results were negative and are the only ones to use pulse mode for photobiomodulation on the reduction of injection pain. So, the exact findings in terms of application time effects and results are difficult to interpret. In this study, 20 sec was used as the minimal application time based on existing literature. Variations in laser parameters, including wavelength, power, application mode, exposure time, tissue type and tissue condition, can affect the outcome [15].

## Conclusion:

Photobiomodulation therapy (PBMT) is a can be used with anesthetic approach. It reduces the prick pain during administration of IANB and thus builds the trust and cooperation with children. It should be that various application parameters such as injection techniques and individual factors (*e.g.*, age) impact photobiomodulation effectiveness in reducing injection pain should be assessed using a large sample size.

## Figures and Tables

**Table 1:** Descriptive statistics for three metrics: the wong-baker faces pain rating score, flacc scale score, and pulse oximeter reading

**Treatment**	**Measure**	**Mean**	**Median**	**Std. Dev**	**Min**	**Max**
Low Level Laser Therapy	Wong-Baker FACES Pain Rating Scale	3	2	1.41	2	6
	FLACC Scale	1.3	0	1.79	0	6
	Pulse Oximeter	84.63	82	10.34	70	112
Placebo	Wong-Baker FACES Pain Rating Scale	4.12	4	2	0	8
	FLACC Scale	2.6	2.5	2.06	0	6
	Pulse Oximeter	90.1	85	11.95	74	120
